# Lower blood phenylalanine concentrations may be of clinical benefit in adults with phenylketonuria

**DOI:** 10.1016/j.ymgmr.2026.101362

**Published:** 2026-09-17

**Authors:** C.M.A. Lubout, A.M.J. van Wegberg, E. van Adrichem-van Dam, M.E. Jansen, E.M. van Steenis, M.R. Heiner-Fokkema, F.J. van Spronsen

**Affiliations:** a Division of Metabolic Diseases, University of Groningen, University Medical Center Groningen, Beatrix Children's Hospital, the Netherlands; b Department of Laboratory Medicine, Laboratory of Metabolic Diseases, University of Groningen, University Medical Center Groningen, the Netherlands

**Keywords:** Phenylketonuria, Phenylalanine levels, Sepiapterin, Sapropterin, Functioning

## Abstract

**Background:**

Treatment in phenylketonuria (PKU) is aimed at lowering blood phenylalanine (Phe) levels, however the target blood Phe levels in adults with PKU are a matter of ongoing debate.

**Objective:**

To describe the experience of adults with PKU regarding mood and self-reported functioning during a period with higher and/or lower blood Phe levels than they were used to during an interventional trial with Sepiapterin.

**Methods:**

Eight adults with PKU were included in an open label interventional trial with Sepiapterin (Sephience®, Phase 3 APHENITY Extension Study NCT05166161). Per protocol, participants who were receiving Sapropterin (*N* = 4) had a washout week before starting with Sepiapterin. The experience of the participants concerning their functioning and feelings were described during the first weeks of the trial when their blood Phe levels changed to higher than usual (in *N* = 4 during a Sapropterin washout week) and to lower than usual (*N* = 8) during treatment with Sepiapterin.

**Results:**

Participants reported worse functioning when their blood Phe levels were higher than usual and improved functioning when their blood Phe levels were lower than usual.

**Conclusions:**

Adults with PKU may experience worsened self-reported functioning at higher blood Phe levels and improved functioning when Phe is lower than their usual blood Phe levels. Therapies that reduce blood Phe levels without the need of a strict diet offer the possibility to objectively study the impact of lower blood Phe levels in adults with PKU and may eventually facilitate individualized target blood Phe levels.

## Highlights

• Adults with PKU may experience worsened functioning at higher blood Phe levels.
• Adults with PKU may experience improved functioning at lower blood Phe levels than usual.
• Therapies are needed that lower blood Phe levels without the burden of diet.

## 1 Introduction

Phenylketonuria (PKU; OMIM # 261600) is an inborn error of phenylalanine (Phe) metabolism, caused by pathogenetic variants in the gene encoding phenylalanine hydroxylase (PAH, EC 1.14.16.1). PAH is responsible for the conversion of Phe to tyrosine, requiring the co-substrate tetrahydrobiopterin (BH4). Untreated PKU leads to severe intellectual disability, epilepsy and psychiatric and movement issues. Extracerebral symptoms include light pigmentation of skin, eyes and hair, eczema and a musty odor. [1] Increased brain Phe levels are considered the most important cause of the clinical picture of PKU. As blood and brain Phe concentrations correlate strongly, blood Phe concentration is the accepted biomarker for monitoring metabolic control. [1], [2], [3], [4] The cornerstone of treatment is lowering of blood Phe with a Phe-restricted diet, a diet low in natural protein, supplemented with Phe-free protein substitutes and special low protein food. [3] However, this diet is considered to be burdensome and challenging to maintain especially in adulthood. [5], [6], [7], [8] Sapropterin (dihydrochloride), an oral synthetic form of BH4, the natural co-factor for the PAH enzyme, can alleviate this strict diet by improving residual PAH activity. However, only a subset of people with PKU respond to sapropterin. [1], [3], [8]

Despite the merits of early and continued treatment in PKU, with neurocognitive outcome in most early-treated individuals in normal range, there are still concerns in adults with early-treated PKU (ET-PKU) regarding neurocognitive performance, social functioning and mental health. [3], [9], [10], [11], [12], [13] Outcomes relate to both historical (lifetime) blood Phe, and current blood Phe concentrations as well as their variation. [14], [15], [16]

The recommended blood Phe target range for adults with PKU remains a topic of ongoing debate. [3], [4], [17], [18], [19] In a randomized controlled trial by Trepp et al., adults with PKU were exposed to a period of higher Phe intake, resulting in increased blood Phe levels. However, no effect was described on different domains of functioning except for sustained attention. It was argued that this effect should be weighted against the burden of a very strict diet. [20] In contrast, in another recent study, Manti et al. found improvement in functioning and IQ in women with PKU who had intensified dietary treatment with target blood Phe < 240 μmol/L due to pregnancy planning. [21] In addition, there are (partly unpublished) reports suggesting that with the treatment with pegvaliase (an enzyme substitution therapy that is given daily by subcutaneous injections), adults with PKU achieving physiological blood Phe concentrations feel much better and experience improved focus. Reports also describe the disappearance of brain fog and headache, as well as less mood disturbances, while recurrence of these symptoms was noted when pegvaliase was stopped for various reasons. Of note, pegvaliase is not a widely available treatment for PKU as it is not reimbursed in all countries and pegvaliase can induce immunological reactions. [3], [6], [22], [23]

Sepiapterin is a synthethic precursor of BH4 that is taken orally and can increase residual PAH activity. Sepiapterin was granted marketing authorization in Europe, Australia, Canada and USA in 2025 and approval application is ongoing in other countries. [8], [24], [25], [26], [27] The objective of this study was to capture the experiences of adults with PKU during the initial weeks of an open label trial (Sephience®, phase 3 APHENITY Extension Study NCT05166161) with PTC923 (sepiapterin) when their blood Phe levels changed from their usual levels. It was hypothesized that both increases and decreases in blood Phe relative to their usual blood Phe levels could give rise to changes in mood and functioning.

## 2 Methods

### 2.1 Participants

Eight adults with PKU, treated in the Dutch center of expertise for PKU and Tyrosinemia at the University Medical Center of Groningen, the Netherlands were included in an open label study involving sepiapterin (Phase 3 APHENITY Extension Study NCT05166161) after written informed consent. The study was approved by the institute review board of the University Medical Center of Groningen (METc 2022.452).

Clinical details of participants are depicted in Table 1. All participants, except participant (P) 4, had an early diagnosis of PKU following newborn screening. Newborn screening allows early diagnosis and pre-symptomatic treatment of PKU. [1], [3] P4 was born before the introduction of newborn screening. He was diagnosed at 2.5 year of age secondary to developmental delay.

**Table 1:** Molecular and clinical characteristics of the participants.

P	Age year (Y)	M/F	PAH nucleotide aberration/ variant name	GPV score^a^	Prescribed natural protein (grams/day)	Prescribed protein equivalent from protein substitute (grams/day)	Sapropterin use and dosage
1	20	M	c.194 T > C; p.Ile65Thr	1.1	22	60	Yes, since age 9 Y. 12 mg/kg/day
			c.1315 + 1G > A; p.IVS12 + 1G > A				
2	36	M	Homozygous c.782G > A; p.Arg261Gln	1.6	20	60	No^b^
3	30	M	c.168 + 5G > C; p. IVS2 + 5G > C	0	17	60–80	No^b^
			c.727C > T; p.Arg243*				
4	55	M	c.829 T > G; p.Tyr277Asp	0.4	11	71	No^c^
			c.1315 + 1G > A; p.IVS12 + 1G > A				
5	24	M	c.194 T > C; p.Ile65Thr	1.1	22	60	Yes, since age 12 Y. 17 mg/kg/day
			c.842 + 1G > A; p. IVS7 + 1G > A				
6	29	M	c.1241 A > G; p.Tyr414Cys	5.1	No diet	–	Yes, since age 16 Y. 16 mg/kg/day
			c.1315 + 1G > A; p.IVS12 + 1G > A				
7	43	F	c.721C > T; p.Arg241Cys	5.5	16	55	No^d^
			c.782G > C; p.Arg261Pro				
8	26	F	c.1315 + 1G > A; p. IVS12 + 1G > A	1.5	12	55	Yes, since age 16 Y. 16 mg/kg/day
			c.1042C > G; p.Leu348Val				

a GPV score was based on the definition described in Hillert et al. with the APV scores of the different alleles mentioned in the supplemental data of this article. [28].

b unresponsive, based on both 48 h and prolonged BH4 loading and a trial period with sapropterin.

c unresponsive, based on both 48 h BH4 loading and prolonged BH4 loading.

d responsive at BH4 48 h loading.

Ages varied from 20 to 55 years (mean 33 years), six male, two female participants. Based on the definition of the severity of PAH deficiency using Genotype Phenotype Value (GPV), six participants had the most severe deficiency (often referred to as classical PKU) with a GPV of 0–2.7. Two participants had a less severe deficiency (often referred to as moderate PKU) with a GPV of 2.8–6.6. [28] Before the start of the trial, seven out of eight participants were treated with a protein restricted diet and protein substitutes, three of them were also treated with sapropterin (P1, 5 and 8). P6 was treated with sapropterin and had an unrestricted diet.

### 2.2 Study protocol and data collection

According to the study protocol (Phase 3 APHENITY Extension Study NCT05166161), participants continued their diet initially without change. Participants on sapropterin (P1, 5, 6 and 8) had a washout week before start of sepiapterin at a dose of 60 mg/kg/day (orally). If the mean blood Phe (measured at the central laboratory) during the first month of sepiapterin treatment was <360 μmol/L, participants proceeded to the Phe-tolerance arm of the study with gradual increases in dietary Phe intake thereafter. [27]

In addition to objectives according to the study protocol, participants were asked to note every change in their mood, behavior, energy level, headache, concentration or other unexpected changes. Participants were contacted weekly by the study team to document the participants' experiences in the participants' own words. The experiences during the first 4–6 weeks of sepiapterin treatment (before proceeding to Phe tolerance arm with dietary adjustment) were included in this study.

### 2.3 Phe measurements

Participants took blood samples by fingerprick to measure Phe concentrations in both volumetric absorptive microsampling by high-performance liquid chromatography-tandem mass spectrometry (HPLC-MS/MS) in a central laboratory (per study protocol, results not included in this study) and in dried blood spot as standard in our laboratory for metabolic diseases. For the dried blood spot analysis in our university laboratory for metabolic diseases, high-performance liquid chromatography (LC20; Shimadzu, Kyoto, Japan) coupled to a triple quadruple mass spectrometer with an electrospray ionization source (API-3200, SCIEX, Framingham, MA, USA) was used. [29], [30] Measurement of dried blood spot Phe levels in our university laboratory for metabolic diseases was included to enable more rapid detection of extreme variations in blood Phe, given possible delays in receiving the per-protocol results from the central laboratory.

Dried blood spot Phe results from the sapropterin washout week (when applicable) were available some days after the start of sepiapterin. Because these blood spots were collected at home and submitted in a batch, both participants and the treatment team were unaware of the actual blood Phe levels during the washout week when the participants reported on their experiences.

### 2.4 Statistical analysis

Historical blood Phe levels during the year before inclusion, blood Phe levels during the sapropterin washout week (when applicable) and the first month of sepiapterin treatment, measured at our university laboratory for metabolic diseases, were included for analysis. Mean or median blood Phe levels, depending on whether the data were normally distributed (tested by a Shapiro-Wilk test) were analyzed for the year before inclusion and during the first month of sepiapterin treatment (IBM SPSS statistics version 28).

## 3 Results

### 3.1 Blood Phe levels

The (mean/median) blood Phe levels during the year before inclusion, course of blood Phe levels during sapropterin washout week (when applicable) and (mean/median) blood Phe levels during the first treatment month with sepiapterin are depicted in Table 2.

**Table 2:** Blood Phe (μmol/L) before start of sepiapterin, during sapropterin washout week (when applicable) and during first month of sepiapterin.

	Mean/median Phe year before inclusion^a^	Phe at start of the washout week	Phe max during washout week	Mean/median Phe during sepiapterin (first month)^a^	Decrease in mean/median Phe with sepiapterin compared to the year before inclusion
1	Mean 356 (± 140)	494	819	Mean 204 (±192)	152 (43%)
2	Mean 572 (± 81)		–	Mean 242 (±46)	330 (58%)
3	Mean 903 (± 136)		–	Mean 728 (±230)^d^	175 (19%)
4	Mean 720 (± 93)		–	Mean 658 (±117)	62 (9%)^e^
5	Mean 409 (± 141)	330	799	Mean 149 (±129)	260 (64%)
6	556 at inclusion^b^	626	925	Median 324 (IQR227–432)	232 (42%)
7	Median 416^c^ (IQR NA)		–	Median 154 (IQR149–176)	262 (63%)
8	Mean 650 (± 161)	573	833	Mean 215 (±50)	435 (67%)

a Mean + SD in case of normally distributed data, median + IQR if not normally distributed.

b no historic values available, value at inclusion is used.

c only 2 historical values available.

d this participant stopped after 21 days, mean from this period.

e in the central laboratory a ≥ 15% Phe decrease was seen compared to the value at screening (sepiapterin responsive according to the study protocol) [27].

During the first sepiapterin treatment month, there was a 9–67% decrease in mean/ median blood Phe levels compared to the year before inclusion. Six participants (P1, 2, 5, 6, 7 and 8) showed a blood Phe <360 μmol/L in the first month on sepiapterin. P3 and 4 only showed some decrease in blood Phe levels during sepiapterin, however not to blood Phe concentrations <360 μmol/L.

### 3.2 Self-reported functioning and blood Phe levels during sapropterin washout (when applicable) and sepiapterin treatment, described per participant

P1 was on sapropterin when entering the trial. During the washout week he noted difficulties in concentration and memory. His family noted he reacted slower than usual, he had difficulty keeping up with conversations and was emotionally different with serious anger outbursts he had never shown before. After starting sepiapterin these problems quickly resolved. In addition his concentration improved even more compared to sapropterin treatment and enhanced social interaction was noted.

During the sapropterin washout period, blood Phe levels rose from 494 to a max of 819 μmol/L. After starting sepiapterin, blood Phe levels quickly dropped, with a mean blood Phe of 204 μmol/L during sepiapterin compared to a mean blood Phe of 356 μmol/L the year before.

P2 had shown some responsiveness on prolonged sapropterin loading, however, natural protein intake could not be increased with sapropterin, so this was discontinued. [31] Initially, he noted no differences while receiving sepiapterin, but his wife reported him as being more calm and less agitated. After 10 days he reported increasingly abdominal pain and constipation which eventually led him to stop sepiapterin after 60 days. After having stopped, he reported that he had experienced an improvement in concentration, calculating skills and that he found it easier to start conversations during sepiapterin treatment.

Blood Phe levels quickly decreased to a mean of 242 μmol/L during sepiapterin compared to a mean blood Phe of 572 μmol/L the year before.

P3 had previously been found unresponsive to sapropterin and was treated with diet therapy. Blood Phe levels were above target range. He had a history of fatigue and frequent headaches. Within a few days after starting sepiapterin he reported feeling different and had no headaches anymore. The second week he noted to have more energy, more focus and to be less stressed. In the third week there was an increased bleeding tendency (spontaneous hematomas and prolonged bleeding after fingerprick) with cold extremities. Because a possible relation with sepiapterin could not be excluded, sepiapterin was stopped after 21 days. The headaches, lack of energy and concentration problems quickly reoccurred. He mentioned difficulties with keeping focus, for example in conversations. He reported a feeling of weak muscles and apathy. Immunological and hematological work-up were unremarkable. Later in the clinical course, the hematomas were considered potentially associated with inhaled medication (formoterol/beclometasone).

After the start of sepiapterin, blood Phe level slowly decreased to a mean blood Phe of 728 μmol/L during sepiapterin with the lowest blood Phe of 472 μmol/L at day 21 compared to a mean blood Phe of 903 μmol/L the year before.

P4 was had previously been found sapropterin unresponsive and was treated with diet therapy. After several weeks of sepiapterin he reported some improvement in concentration, and felt more more clear headed and less fatigued. His blood Phe levels slowly decreased to mean 658 μmol/L with sepiapterin compared to a mean blood Phe of 720 μmol/L the year before.

P5 was treated with sapropterin and diet. From the third day of the washout week, he noted progressive problems with concentration. Reading was more difficult. During that week he felt emotionally different, labile. He was more fatigued and had headaches. The day after starting sepiapterin, he reported these symptoms improved and he could study better.

Blood Phe increased from 330 μmol/L to a maximum of 799 μmol/L during the washout week and quickly went down after starting sepiapterin to a mean of 149 μmol/L during sepiapterin compared to a mean of 409 μmol/L the year before.

P6 received sapropterin therapy and reported an unrestricted diet. Blood Phe was monitored infrequently, with a concentration usually around 600 μmol/L. At the end of the sapropterin washout week, he noted headaches, problems concentrating, irritability and fatigue. He also reported to experience issues at work. During the first 2–3 weeks after starting sepiapterin these symptoms persisted, in addition he felt ill. Thereafter, symptoms slowly improved but he did not feel as well as under sapropterin, although this could also be related to an intensive period at work.

At the end of the washout week, his blood Phe increased from 556 μmol/L at inclusion to a max of 925 μmol/L. After start of the sepiapterin, blood Phe dropped to a median of 324 μmol/L.

P7 was responsive to sapropterin but decided not to start sapropterin and was treated with diet only. After the first week of sepiapterin she indicated that the tension she normally felt around her left eye, notably when blood Phe was >450–500 μmol/L, had decreased. She reported illness diagnosed as flu and a pneumonia after two weeks of sepiapterin. During this illness she felt low in energy, afterwards this improved.

After starting sepiapterin, median blood Phe fell to 154 μmol/L. Blood Phe the year before was measured infrequently with a median of 416 μmol/L.

P8 received sapropterin therapy with a Phe-restricted diet. During the washout week she reported a decline in concentration (at work, with conversations) and more brain fog. She recognized this from periods when blood Phe levels were higher. After start of sepiapterin, she noticed improved concentration, more alertness and she could more easily participate and react in conversations.

During the washout week blood Phe increased from 573 umol/L to a maximum of 833 μmol/L. After start of sepiapterin, blood Phe decreased to a mean of 215 μmol/L during sepiapterin compared to a mean blood Phe of 650 μmol/L the year before.

## 4 Discussion

### 4.1 Discussion

This is the first report describing the experience of eight individual adults with PKU when their blood Phe levels changed from their usual range during an interventional study with sepiapterin.

The four participants receiving sapropterin therapy (P1,5,6,8) showed, as expected, an increase in blood Phe above their usual levels during the washout period. They (or members of their social circle) reported progressive problems in functioning (headache, fatigue, concentration, mood) during the washout week. These symptoms were the most severe in the participants (P1 and 5) whose blood Phe levels were usually within treatment range (< 600 μmol/L) [3] with a rmore pronounced increase in blood Phe measured during the washout week. After starting sepiapterin these symptoms rapidly disappeared as blood Phe levels decreased in all but P6 (possibly related to infection and stress at work).

Six participants (P1,2,3,5,7,8) reported clear improvements in self-reported functioning (improved concentration, improved conversations, less brain fog, fatigue and headaches) after starting sepiapterin, in two the effect was less pronounced (P4 and 6). P4 showed only little improvement in blood Phe levels. In P6, blood Phe was lower compared to his usual blood Phe levels but possibly other factors played a role as indicated before. The relative change in blood Phe levels with a strong decrease in blood Phe levels compared to the period before receiving sepiapterin in most of the other six participants may explain the more notable effect seen in them.

The primary cause of symptom changes noted is unclear. It could be explained by the actual or relative change in blood Phe concentration, or reaching a certain threshold. Therefore, the question remains whether the effective treatment range may differ between individuals with PKU. [11], [16]

Possibly, sepiapterin does not only decrease blood Phe, but may also have an effect on symptoms due to a direct effect on cerebral neurotransmitter metabolism. A possible neurotransmitter effect may explain the discrepancy in P3, where clinical improvement seemed discrepant to the reduction in blood Phe levels. P3 noted a remarkable improvement in functioning and mood after start of sepiapterin, even before blood Phe was <600 μmol/L. From sapropterin it is known that it can cross the blood-brain barrier at a dose of at least 20 mg/kg but even much higher doses are probably needed to have a neurotransmitter effect. [32] It was demonstrated that sepiapterin can cross the blood-brain barrier at a dose of 60 mg/kg (as used in the participants described in the presented study) resulting in increased BH4 levels in CSF in healthy individuals. [33] Whether sepiapterin can have an effect on cerebral neurotransmitters in PKU, remains to be elucidated.

To summarize, participants reported a worsened mood state and self-reported functioning during sapropterin washout when their blood Phe levels were higher than usual. This is in line with the findings by Hoedt et al., who showed a negative effect of higher blood Phe on mood and sustained attention. As some of the participants in the trial of Hoedt et al. showed a baseline blood Phe above the upper target, the effect might even be more pronounced if blood Phe levels increased from within treatment range to elevated levels as observed in most of our participants during the washout week. [34]

The findings of our study seem in contrast with the findings by Trepp et al. In their study (double-blind, placebo controlled, crossover design), an increase of Phe intake in adult participants with PKU was reported to have no effect on different domains of functioning except for sustained attention. They noted that the significant effect on sustained attention may have been influenced by an improved performance in the placebo group. The difference between the findings by Trepp et al. and our study might be explained by the higher baseline blood Phe levels in the study by Trepp et et al. (median 749 range 380–1208 μmol/L) compared to blood Phe levels in our study (median blood Phe year before inclusion 564 range 356–903 μmol/L). There could be a possible plateau effect of higher blood Phe levels in the study by Trepp et al., with no additional effect seen when blood Phe was further elevated. [20]

Based on the same study, Hauri et al. reported that adults with PKU were unable to subjectively identify the period of high blood Phe levels, suggesting that non-specific symptoms such as altered cognition should not be related to blood Phe levels. However the subgroup correctly indentifying the period of elevated blood Phe had a significant higher blood Phe level with an larger increase in blood Phe level. [35] This is in line with our observation that participants (P1 and P5) with most evident change in self-reported functioning, went from blood Phe <600 μmol/L to much higher blood Phe during sapropterin washout and then decreased to even lower blood Phe (<360 μmol/L) on sepiapterin.

Participants in our study described improvement in mood and self-reported functioning when blood Phe levels decreased to below usual levels. Despite previous belief that they were functioning at optimal levels, they reported awareness of improvement in functioning. In the study by Manti et al., in which adult women with PKU planning pregnancy had intensified dietary treatment with target blood Phe < 240 μmol/L, an improvement in functioning and IQ was shown, however, it took 3–6 months <240 μmol/L before a maximal effect was reached. [21] The presented cases show that it may take less time to see a difference in self-reported functioning, maybe due to the sharp decrease in blood Phe as seen in some. In addition, in the study of Manti et al., compliance with the more strict dietary restrictions required for pregnancy may have lessened positive perceptions of improved functioning allowed by lower blood Phe. [7], [21] For a short period of time (while planning to become pregnant) this intensified diet might be feasible, but maintaining this strict diet at the long term remains challenging. [5], [6], [8], [21]

In a study of pegvaliase treatment, improved neuropsychiatric symptoms were described alongside lower blood Phe levels. However, in this study, also Phe tolerance was increased, not discriminating between an effect of lowering blood Phe and liberalizing diet on this improvement. [22] The strength of our study is that the self-reported functioning is described for the period that blood Phe levels were lower than usual but with no change in dietary restriction yet.

There is professional disagreement concerning the most appropriate treatment range for blood Phe in adults with PKU. [36] European guidelines recommend <600 μmol/L [3], while United States guidelines recommend <360 μmol/L. [4] The study by Manti et al. suggested <240 μmol/L as most effective. [21] There is also discussion about whether target blood Phe levels in adults should be relaxed or even abandoned. The debate includes that some adults with ET-PKU seem to live happy and fulfilled lives, even with blood Phe >600 μmol/L. [18], [19] Some adults with PKU report no improvement in functioning when blood Phe decreases, but typically their blood Phe concentrations are then still above 600 μmol/L, due to the difficult compliance with diet. [5], [6], [8]

Although the treatment range for blood Phe below 240 μmol/L in the study by Manti et al. reflected tighter control recommended for pregnancy, both the results of the study of Manti et al. and the presented study suggests that adults with PKU may experience functional benefit with lower than usual blood Phe levels. To determine whether adults with PKU function better at (supra)physiological blood Phe concentrations there is a need for treatments that makes low blood Phe concentrations possible without the burden of a very strict diet that is difficult to maintain. [6], [8] In addition, interindividual variability in Phe sensitivity may also play role. [4], [9], [15], [16] Therefore, in the future, more individually tailored target blood Phe levels may be adapted rather than a one size fits all guideline. [11], [15].

### 4.2 Limitations

The experience of change in mood and self-reported functioning described by the participants were not standardized by means of a validated questionnaire. However, there was striking similarity in what participants reported during moments of higher and lower blood Phe. The described reports give an insight in what the participants feel and correspond to the observations described in literature like perceived differences in concentration, fatigue, emotion, behavior, headache and brain fog. [3], [34], [37], [38]

In the earlier discussed report by Hauri et al., it is mentioned that expectations of participants might play role in self-reported symptoms. [35] In our study, expectations might have played role as well. At the same time, in most participants symptoms clearly occurred with a rise in blood Phe and improved alongside a decrease in blood Phe. In addition, participants and study team were not aware of the actual blood Phe results at time of the washout week and first days of sepiapterin treatment when self-reported functioning was documented. However, participants were not blinded for medication intake, so a placebo effect of the study medication and nocebo effect of the washout week might play a role.

In this study, only the experiences from the initial weeks following the start of sepiapterin were documented, during the period in which dietary treatment had not changed. We acknowledge that additional positive experiences were reported later in the course of the study. It is possible that a longer observation period is required to adequately capture all these experiences.

The number of studied participants was low, the experience in a larger group of participants would give more information on how blood Phe levels can affect functioning and the interindividual variability between adults with PKU.

Despite limitations, the findings of our pilot study suggests the need for further study. Future studies should preferably examine the effect of changes in blood Phe concentrations in a larger cohort of adults with PKU, ideally blinded to their blood Phe concentrations, while also paying attention to the effect of changes in diet on functioning.

## 5 Conclusion

Eight adults with PKU described their functioning at different blood Phe levels with problems in self-reported functioning in participants with higher blood Phe levels than usual. An improvement in functioning was described when the blood Phe levels were lower than usual, with the most pronounced effect in participants with blood Phe levels well <360 μmol/L. As these improvements were unexpected for both the participants and their social network, adults with PKU may not always realize that further improvement could be possible. Therefore, we should consider to (initially) aim at low blood Phe levels to give adults with PKU the possibility to decide for themselves their optimal, individualized target blood Phe level. This underscores the need for therapies that help lower blood Phe levels without the burden of a strict(er) diet. Eventually, a consensus between guidelines from different countries is needed.

## Details of ethics approval

The study was approved by the institute review board of the University Medical Center of Groningen (METc 2022.452).

## A patient consent statement

All participants provided written informed consent to participate in Phase 3 APHENITY Extension Study NCT05166161 and for publication of this manuscript.

## CRediT authorship contribution statement

**C.M.A. Lubout:** Writing – original draft, Methodology, Investigation, Formal analysis, Conceptualization. **A.M.J. van Wegberg:** Writing – review & editing, Investigation, Conceptualization. **E. van Adrichem-van Dam:** Writing – review & editing, Investigation. **M.E. Jansen:** Writing – review & editing, Project administration, Investigation. **E.M. van Steenis:** Writing – review & editing. **M.R. Heiner-Fokkema:** Writing – review & editing, Resources. **F.J. van Spronsen:** Writing – review & editing, Supervision, Investigation, Conceptualization.

## Funding

This research did not receive any specific grant from funding agencies in the public, commercial, or not-for-profit sectors.

## Declaration of competing interest

The authors declare the following financial interests/personal relationships which may be considered as potential competing interests: Charlotte Lubout reports a relationship with National PKU Alliance that includes: funding grants. Francjan van Spronsen reports a relationship with National PKU Alliance that includes: board membership and funding grants. Francjan van Spronsen reports a relationship with PTC Therapeutics Inc. that includes: board membership and consulting or advisory. Francjan van Spronsen reports a relationship with BioMarin Pharmaceutical Inc. that includes: board membership, consulting or advisory, funding grants, and speaking and lecture fees. Francjan van Spronsen reports a relationship with Nutricia Ltd. that includes: funding grants and speaking and lecture fees. Francjan van Spronsen reports a relationship with Vitaflo International Ltd. that includes: funding grants and speaking and lecture fees. Francjan van Spronsen reports a relationship with Metakids Foundation that includes: funding grants. Francjan van Spronsen reports a relationship with European Society for Phenylketonuria and Allied Disorders Treated as Phenylketonuria (ESPKU) that includes: board membership and funding grants. Francjan van Spronsen reports a relationship with Nederlandse PKU vereniging that includes: board membership. Francjan van Spronsen reports a relationship with stichting PKU research that includes: funding grants. Francjan van Spronsen reports a relationship with Arla Food Int that includes: board membership. Francjan van Spronsen reports a relationship with AlltRNA that includes: board membership and consulting or advisory. Francjan van Spronsen reports a relationship with Pluvia Biotech that includes: board membership and consulting or advisory. Francjan van Spronsen reports a relationship with Otsuka Pharmaceutical Co Ltd. that includes: speaking and lecture fees. Francjan van Spronsen reports a relationship with Sanofi that includes: board membership. Annemiek van Wegberg reports a relationship with PTC Therapeutics Inc. that includes: board membership and speaking and lecture fees. Annemiek van Wegberg reports a relationship with Nutricia Ltd. that includes: funding grants and speaking and lecture fees. Annemiek van Wegberg reports a relationship with POA Pharma Scandinavia AB that includes: speaking and lecture fees. Ellis van Steenis reports a relationship with BioMarin Pharmaceutical Inc. that includes: funding grants. Esther van Adrichem-van Dam reports a relationship with Nutricia Ltd. that includes: travel reimbursement. Esther van Adrichem-van Dam reports a relationship with Vitaflo International Ltd. that includes: travel reimbursement. Charlotte Lubout, Annemiek van Wegberg, Margreet Jansen and Francjan van Spronsen were as study team involved during the Phase 3 APHENITY Extension Study NCT05166161 (https://clinicaltrials.gov/study/NCT05166161). PTC Therapeutics is the sponsor of this Sephience®, Phase 3 APHENITY Extension Study, but was not involved in the presented study in this manuscript. If there are other authors, they declare that they have no known competing financial interests or personal relationships that could have appeared to influence the work reported in this paper.

## Data Availability

Data described in the manuscript can be made available upon request from the corresponding author pending approval and data sharing agreement.
